# Advancements in Cancer Immunotherapies

**DOI:** 10.3390/vaccines11010059

**Published:** 2022-12-27

**Authors:** Ruchi Roy, Sunil Kumar Singh, Sweta Misra

**Affiliations:** 1 UICentre for Drug Discovery, College of Pharmacy, University of Illinois at Chicago, Chicago, IL 60607, USA; 2 Department of Surgery, University of Illinois at Chicago, Chicago, IL 60607, USA

**Keywords:** tumor microenvironment, cancer vaccines, tumor-infiltrating lymphocytes, combination therapy, checkpoint-inhibitors, CAR T cell therapy

## Abstract

Recent work has suggested involvement of the immune system in biological therapies specifically targeting tumor microenvironment. Substantial advancement in the treatment of malignant tumors utilizing immune cells, most importantly T cells that play a key role in cell-mediated immunity, have led to success in clinical trials. Therefore, this article focuses on the therapeutic approaches and developmental strategies to treat cancer. This review emphasizes the immunomodulatory response, the involvement of key tumor-infiltrating cells, the mechanistic aspects, and prognostic biomarkers. We also cover recent advancements in therapeutic strategies.

## 1. Tumor Microenvironment in Cancer

Cancer is currently the second leading cause of death in the United States based on the report of Cancer Statistics 2022 [1]. Every year, new cancer cases and cancer deaths are increasing drastically in the United States, causing major economic burdens for patients, healthcare systems, and countries due to healthcare spending, as well as productivity losses from morbidity and premature mortality.

**Figure 1:**
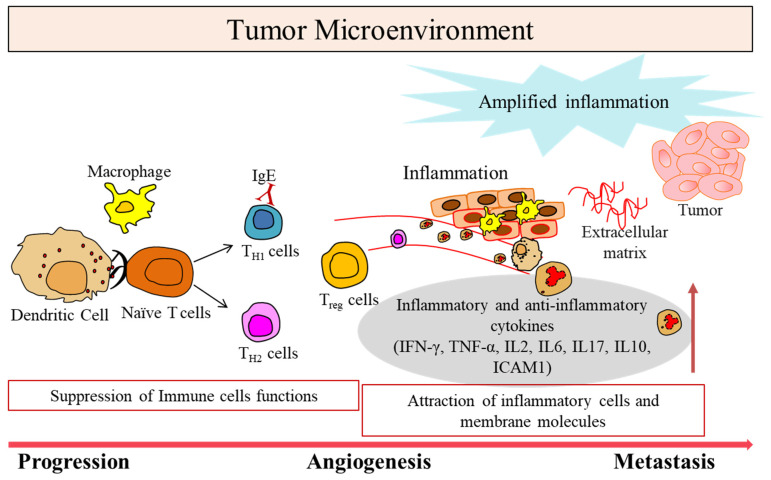
Tumor microenvironment in cancer. Schematic depicting progress of tumor involving suppression of immune function, tumor cells proliferation leading to metastasis.

Cancer development and its progression is critically dependent on the tumor microenvironment (TME), which can affect how a tumor grows and spreads. The dynamic interactions of cancer cells with their microenvironment consist of an abundant fibrous matrix, immunosuppressive cells, and blood vessels, which help to protect the tumor tissue from the immune systems’ trap (Figure 1). This interaction is essential to stimulate the heterogeneity of cancer cells, clonal evolution, and to increase the multidrug resistance, ending in cancer cell progression and metastasis. Several reports have elucidated their clinicopathologic significance in predicting outcomes and therapeutic efficacy [2,3]. Several studies have established that a dynamic and mutualistic interaction between tumor cells and the surrounding stroma determines the fate of tumor growth. The importance of tumor-related structures, as well as upregulated signaling pathways in both cancer cells and the tumor microenvironment, is well known. Differences in the compositions of resident cell types within the TME, including tumor-associated macrophages (TAMs), cytotoxic T cells (CD8^+^T), helper T cells (CD4^+^T), dendritic cells (DCs), resting fibroblasts, mesenchymal stem cells, and associated inflammatory pathways, have been reported in patients with cancer [4,5,6,7]. TAMs play a crucial role in tumor progression by causing by promoting genetic instability, nurturing cancer stem cells, supporting metastasis, and taming protective adaptive immunity. They typically express characteristic surface molecules, such as the hemoglobin scavenger receptor (CD163) and macrophage mannose receptor 1 (CD206) [4].

Changes in the number and type of immune cells infiltrating TME decide the clinical outcomes in various malignancies such as gastric cancer, melanoma, urothelial cancer, lung cancer, and breast cancer [1,7,8,9,10]. The consequence of such crosstalk is reflected in tumor formation and the immune response [11], as well as chemotherapy benefit [12]. Tumor progression is associated with the generation of signals originating from tumor cells, lymphocytes, and stromal cells. Different subtypes have been characterized according to immune activation responses to viruses, interferon-γ (IFN-γ), transforming growth factor-β (TGF-β), epithelial–mesenchymal transition (EMT), and angiogenesis pathways. These responses are considered as T cell suppressive and may be responsible for significantly worsening the prognosis in cancer.

As a result, a growing awareness of the fact that the success of chemotherapy and radiotherapy relies on the induction of a durable anticancer immune response can help to stabilize well beyond the discontinuation of treatment.

## 2. Immunomodulatory Roles of Lymphangiogenesis in TME

Significant role of lymphatic vessels in the tumor microenvironment is known to promote tumor metastasis in many cancers by undergoing activation, hyperplasia, and lymphangiogenesis in the tumor microenvironment and in the tumor-draining lymph node. Lymphatic vessels were considered passive participants in tumor progression and metastasis by simply providing a physical route for tumor cells dissemination to draining lymph nodes. However, recent studies have highlighted new roles of the lymphatic endothelium in regulating host immunity by identifying several key lymphatic-specific molecular markers, and a complex array of lymphangiogenic factors, chemokines, and immune cell subsets [13]. Accumulation of immune cells during the process of angiogenesis in TME is mediated by several growth factors, such as vascular endothelial growth factor A (VEGFA), PDGF, epidermal growth factor (EGF), IL-6 and IL-8 [14]. Other prolymphangiogenic factors identified were VEGFC and VEGFD, which bind to the receptors present on the lymphatic endothelium such as tyrosine kinase receptors and VEGF receptors (R)–3 [15,16,17,18]. Neuropilin receptor-2 (Nrp-2) is a coreceptor for VEGF receptor signaling by enhancing the binding between VEGF ligands and receptors [19,20]. Specifically, a study showed that both VEGF-A and VEGF-C induce the interaction of NRP2 with VEGFR-2 by enhancing the VEGFR-2 phosphorylation [19]. Nrps play a crucial role in cardiovascular development, axon guidance, tumorigenesis, inflammation, and cardiovascular disease. Nrps can also function as a modulator of endothelial cell migration independently of VEGF receptors [21,22]. VEGFs plays role in modulating T cell proliferation and their phenotypic expression and activity. A study conducted in a mouse mammary tumor model using small interfering RNA-mediated downregulation of VEGF-C showed its impact on tumor-infiltrating lymphocytes and decreased tumoral lymphangiogenesis [23]. VEGFs not only affect lymphocytic activities but are also a key regulator of natural killer (NK) cell cytotoxicity that determines their critical role in mediating immune tolerance [24].

Lymphatic vasculature helps in trafficking of immune cells from peripheral tissues to lymph nodes through the signaling of chemokines, where adaptive immune responses are instigated.

**Figure 2:**
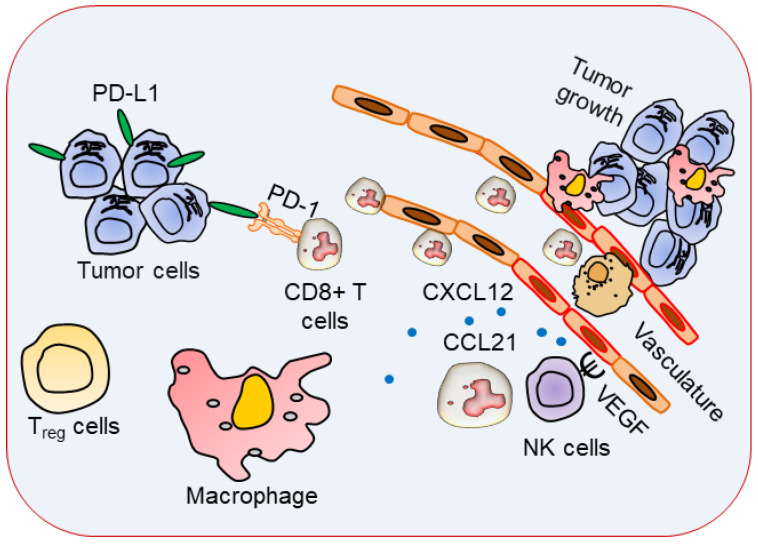
Lymphangiogenesis role in tumor microenvironment. Tumors infiltrated with immune cells with Treg and CD8^+^ effector T cells become functionally inhibited by the effects of PD-L1 expressing tumor cells. Lymphatic vessels expressing chemokine receptors CXCR4 and CCR7 binds to respective ligands CXCL12 and CCL21, leading organ tumors to metastasize.

Chemokines are crucial for providing navigational cues to migrating cancer cells by facilitating tumor growth by promoting EMT as well as angiogenesis. It is generally accepted that the expression of certain chemokine receptors on the surface of the cancer cells promotes metastasis and organ-specific metastasis. For example, the pattern of breast cancer metastasis is largely determined by the interaction between the chemokine receptors on cancer cells and the chemokines expressed. Chemokine receptors CXCR4 and CCR7 ligands (CXCL12 is also referred to as stromal cell-derived factor-1 [SDF-1] and CCL21) are widely expressed on human breast cancer cells and on the organs that tumors normally metastasize [25]. Several reports elucidated that tumor-associated lymphatic vessels, but not normal lymphatic vessels, express a high concentration of CXCL12, highlighting an active role for the tumor-associated lymphatic endothelium in metastatic tumor spread [26,27,28,29] (Figure 2). The chemokines CCL19 and CCL21 ligands for CCR7 are important for active metastatic dissemination of malignant cells via the lymphatic system. This suggests lymphatic vessels and lymphangiogenesis play important immunomodulatory roles in the tumor microenvironment.

## 3. Immune Cells with Specific Phenotypes in TME

Cancer biomarkers help in characterizing alterations in the tumor. A number of genes associated with lymphocyte regulation, cytokine signaling, lymphocyte markers, checkpoint pathways, and tumor characterization have been identified as a predictive biomarker for cancer. The majority of tumors show evidence of a T-cell-infiltrated phenotype. TME and the immune system play critical roles in cancer progression and clinical outcome where regulatory (Treg) and effector T cells’ infiltration contributes to the maintenance of self-tolerance and an immune-homeostasis-creating immunosuppressive environment by suppressing antitumor immunity in the TME. T-cell-inflamed TME is characterized by the elevated expression of type 1 interferon, as well as promigratory chemokines that result in the recruitment of activated CD8^+^ effector T cells into the tumor parenchyma [30]. Anticancer can be segregated into three main phenotypes: the immune-excluded phenotype, the immune-desert phenotype, and the inflamed phenotype. An immune-excluded tumor represents a specific chemokine state that is characterized by the presence of different immune cell types in the aggressive margin or stroma of the tumor but cannot infiltrate into the tumor parenchyma [31,32]. On the contrary, the immune-desert phenotype is caused by immunological ignorance, which is characterized by the absence of appropriate T cell priming or activation in the parenchyma or stroma of the tumors and poor response to immune checkpoint inhibitors treatment [33,34]. Inflamed tumors are highly infiltrated with number of immune cells subtypes, including immune-inhibitory regulatory T cells, myeloid-derived suppressor cells, suppressor B cells, and cancer-associated fibroblasts. It contains proinflammatory cytokines that should provide a more favorable environment for T cell activation and expansion [35]. Therefore, utilizing the role of cytotoxic T cells to kill cancer cells can be an effective immunotherapy. The presence of activated CD8^+^ T cells, both within the tumor and in the peritumoral stroma, has been shown to have significant positive prognostic import [36,37]. A high ratio of CD8^+^ T cells to Foxp3^+^ regulatory T cells (Treg) in the ovarian cancer tumor microenvironment has been associated with a particularly favorable clinical outcome [38]. A study was conducted to identify factors associated with success or failure of checkpoint therapy, in which they performed transcriptomes analysis in immune cells from tumor samples of melanoma patients treated with checkpoint therapy (anti-PD-1, anti-CTLA4+PD-1, and anti-CTLA4). Their results highlighted how the transcription factor 7 (TCF7) is selectively expressed in memory-like T cells, which is a top marker associated with responding lesions. It is a part of the Wnt/β-catenin signaling involved in the differentiation, self-renewal, and persistence of memory in CD8^+^ T cells. Next, they determined the states of immune cells by applying high-dimensional single-cell RNA sequencing (scRNA-seq) because the association of T cell states with clinical responses is well established. They found more TCF7^+^CD8^+^ cells in responding samples, whereas more TCF7^−^CD8^+^ cells were in non-responding samples, which suggests the ratio of CD8^+^TCF7^+^ to CD8^+^TCF7^−^ tumor-infiltrating lymphocytes is strongly correlated with improved response and survival in melanoma patients treated with anti-PD-1 [34,39]. Tumors showing a high expression of PD-L1 and indoleamine-2,3-dioxygenase (IDO) display an increased percentage of CD4^+^CD25^+^Foxp3^+^ Tregs cells [40,41,42]. Spranger et al. suggested that upregulation of PD-L1 and IDO is driven by IFN-γ produced by CD8^+^ T cells in vivo. Furthermore, the accumulation of Treg cells is also CD8^+^ T cell-dependent through the production of the chemokine CCL22 via CCR4 [43,44]. A study conducted by Balatoni et al. on surgical tissue samples from 30 patients with metastatic melanoma treated with ipilimumab evaluated the prognostic and predictive associations of immune cell infiltration and overall survival after treatment. A higher prevalence of several immune cell types including CD4^+^, CD20^+^ B cells, CD134^+^ and CD137^+^ cells, and NKp46^+^ cells was seen, particularly FOXP3^+^ cells and CD8^+^ T lymphocytes [34,45].

A substantial body of evidence suggests that NK cells contribute to host control of hematologic malignancies and contribute to tumor control in solid tumors [44,46,47]. Association between TAMs and anti-PD-1 response has been reported in melanoma cases. It has been established that the association between Fcγ receptor (FcγR), expressed by the host bone marrow cells and Fc domain glycan of the drug, could determine the ability of PD-1-TAMs to capture anti-PD-1 drugs from the surface of T cells, leading to PD-1 inhibitor resistance [48,49]. In addition, anti-PD-1 response also affect cytotoxic T cells, indicating an increase in CD8^+^ T cells and NK cells and a decrease in macrophages [34,50,51].

Recently, a study conducted by Crist et. al. has shown that the antidiabetic agent metformin slows tumor growth and progression in vitro and in combination with chemoradiotherapy in patients suffering from head and neck cancer squamous cell carcinoma (HNSCC) in their phase 1 clinical trial (NCT02325401). It increases the activated peripheral NK cell populations, enhanced HNSCC NK cell cytotoxicity, and inhibited the CXCL1 pathway while stimulating the STAT1 pathway [52]. It also influences anticancer immunity in esophageal squamous cell carcinoma (ESCC) in both humans and mice, via triggering an AMPK activation and STAT3 inactivation. In clinical trials, low-dose metformin recruits a greater number of CD8^+^ cytotoxic T lymphocyte and CD20^+^ B lymphocyte while enhancing tumor-suppressive (CD11c+) and reducing tumor-promoting (CD163^+^) macrophages in TME [53]. In addition to this strategy, mannose-modified, macrophage-derived microparticles (Man-MPs) loading metformin (Met@Man-MPs) were used to effectively target M2-like TAMs to transform them into M1-like phenotypes. This strategy reprogrammed the TME towards an inflamed anti-tumor microenvironment by increasing the recruitment of CD8^+^ T cells. The Met@Man-MPs approach boosted the anti-PD-1 antibody therapy and developed long-term memory immunity [54]. Interestingly, the anti-tumor efficacy of the PD-L1 depression strategy was found to be superior to the conventional anti-PD-L1 therapy in terms of selectivity and efficacy. This group has developed a mitochondria-oxidative phosphorylation (OXPHOS) depression nanosystem using IR-LND (conjugate of mitochondria-targeted heptamethine cyanine dye IR-68 with mitochondrial complexes I and II depression agent lonidamine (LND)) assembled with albumin (Alb) to form IR-LND@Alb nanoparticles [55]. Another promising therapy that has the same limitation of causing severe hypoxia and PD-L1 over-expressed immunosuppressed TME is photodynamic therapy (PDT). A recent study had constructed a MB@Bu@MnO2 nanoparticle system to depress the PD-1/PD-L1 axis with two-step oxygen regulation, where Buformin (Bu, an OXPHOS disrupting agent with PD-L1 depression and oxygen reversion ability), and methylene blue (MB) as a PDT drug with PD-1 inhibition capacity were composed with manganese dioxide albumin (MnO2@Alb) as a carrier of this nanosystem. This resulted in selective delivery to tumor tissues and enhanced T cell infiltration and improved its tumor cell-killing ability, reverting the immunosuppression [56]. PDT not only induces ROS production to damage tumor cells but also promotes the antitumor immunity of T cells through stimulating the production of IFN-γ. Therefore, another composite of metformin (Met) and IR775 in liposome (IR775@Met@Lip) had been studied to solve this problem. The IR775@Met@Lip was found to reverse tumor hypoxia by enhancing ROS production to elicit more damage and downregulate PD-L1 [57].

The drawback of regular chemotherapy is increased in the expression of PD-L1 in almost all kinds of cancers, causing reduced efficacy of T-cell-mediated immune killing in tumors. Recently, chitosan oligosaccharide (COS), a biomaterial derived from the N deacetylation product of chitin, has been found to inhibit the upregulated PD-L1 expression. Due to the property of COS to significantly restrict the growth of CT26 tumors, it has been combined with Gemcitabine (GEM), one of the typical chemotherapeutic drugs, leading to a more remarkable tumor remission [58].

In conclusion, targeting immune cells in TME could be a great predictive biomarker for immune checkpoint inhibitors.

## 4. Cancer Immunotherapy

**Figure 3:**
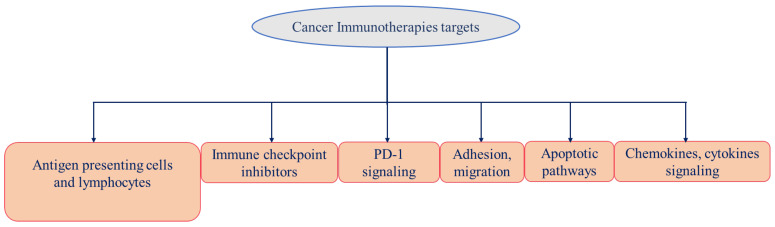
Promising therapies are being developed, targeting immune cells to fight cancer.

Cancer immunotherapies involving T lymphocyte have become a primary goal for engaging the immune system in fighting cancer (Figure 3). Therefore, recent studies are emphasizing the ability of T cells to promote cancer treatments involving checkpoint blockade, adoptive cellular therapy, and cancer vaccinology [59,60,61,62]. Cancer vaccines boost the immune system to mount an attack against cancer cells (see the current status of cancer immunotherapies available in Table 1 and Table 2). By default, the immune system responds chemotactically to the known or self-substance to work normally in our system, whereas it signals a danger when it encounters a non-self or foreign substance. Cancer cells express certain molecules called cancer-specific antigens, neoantigens, or tumor associated antigens (TAAs) on their surface that healthy cells do not express. These TAAs are recognized by cytolytic T lymphocytes (CTL). A melanoma-associated antigen, MZ2-E, which is a rejection antigen that is recognized by the patients’ autologous, tumor-directed and specific cytolytic, CD8^+^ CTL [63]. The association of the antigen MZ2-E with the HLA-A1 molecule was confirmed by a nonapeptide which was recognized by CTL when it was presented by mouse cells transfected with an HLA-A1 gene [64]. The advantage of a higher expression of TAAs on tumor cells and minimally on normal tissues can be utilized in creating therapeutic vaccine-based approaches [63]. Boosting immune effector mechanisms to specifically target cancer cells may be utilized to inhibit the further growth of advanced cancers. Historically, vaccination approaches in the 1970s were based on autologous tumor vaccines prepared using patient-derived tumor cells. These tumor cells were irradiated and administered together with an adjuvant or virus to the individual from whom the tumor cells were isolated to stimulate immune responses [65]. Unfortunately, this approach faces multiple limitations due to nonavailability of tumor specimens, most notably in non-small cell lung cancer (NSCLC) [66]. Vaccines should fulfill the criteria of eliciting more robust immune responses without causing autoimmune-related toxicities. Therefore, it is urgent to find newer approaches to progress cancer treatment better, with high efficacies and enhanced overall survival.

## 5. Personalized Recombinant Cancer Vaccines

Cancer immunotherapy becomes an indispensable component of cancer treatment by targeting TAAs. It has achieved multiple clinical benefits and does not induce unwanted off-target effects.

Therefore, these developments enforce the making of personalized recombinant vaccines that have high efficacy and few side effects in cancer immunotherapy. Surprisingly, there is a high number of mutation burden within tumor types, ranging from 10 s to 1000 s of mutations [81]. Higher mutational events in the tumor have been correlated with greater immunogenicity and survival after checkpoint blockade treatments, e.g., ipilimumab and tremelimumab are antibodies against cytotoxic T-lymphocyte antigen 4 (CTLA-4) treatment prolonged overall survival in patients with melanoma [82,83]. However, those cancer types with fewer mutations, such as pancreatic cancer, do not respond well to immunotherapies. Interestingly, only few gene variants that encode peptides that are specific to TAAs spontaneously generate immune responses [84]. TAA-induced cytokine responses are dependent on selective binding to MHC class II that suggests the role of CD4^+^ T cells rather than CD8^+^ T cells [85,86]. However, several studies have demonstrated the role of CD4^+^ and CD8^+^ T cell responses to TAA vaccines in various cancer types [86,87,88,89,90]. To identify which tumor-derived peptides could potentially form a suitable TAA with the patient’s MHC alleles is assessed by prediction algorithms. Nonetheless, greater precision and effectiveness could be achieved by further research and technological developments to better understand the mechanisms of antitumoral immune responses. To address this aspect, various advancement in immunotherapeutic approaches need to be made.

## 6. Combination Therapies

In recent years, there has been a steep rise in the development and implementation of combination therapies. This therapy couple agents with distinct mechanisms of action have augmented treatment success in various cancers and have improved patient survival. The two most potent examples of T cell immune checkpoint molecules, anti-CTLA-4 and anti-programmed cell death protein 1 (PD-1) antibodies, provide a synergistic effect to regulate antitumoral immunity in a complementary manner that has revolutionized cancer treatment [59,60]. A number of studies have shown that both CTLA-4 and PD-1 checkpoint inhibitors have resulted in increased patient survival for a wide variety of recalcitrant cancers such as melanoma, renal cell carcinoma, squamous cell carcinoma, and non-small cell lung cancer compared to conventional chemotherapies [91,92].

## 7. Immune Checkpoint Inhibitors

The recent development of immune checkpoint inhibitors (ICIs) has revolutionized cancer treatment and has improved patient survival. ICIs specific for checkpoint proteins, such as CTLA-4 or PD-1, have been approved for the treatment of several cancer types, including non-small cell lung cancer (NSCLC), melanoma, head and neck cancer, bladder cancer, and renal cell cancer. “Checkpoint” proteins on immune cells act like switches that need to be turned on or off to keep immune responses in check. Checkpoint inhibitors help the immune system to attack the cancer cells indirectly by keeping T cells from killing tumor cells. A PD-1 expression on T cells helps them to act as a type of “off switch” that keep T cells from attacking other cells in the body by binding to a programmed death ligand 1 (PD-L1) cell. This way, the binding of PD-1 to PD-L1 signals to T cells to leave the other cell alone. But some cancer cells escape the immune attack by expressing large amounts of PD-L1. Given that multiple studies in a variety of tumors have demonstrated that among immunotherapies, the PD-1 and PD-L1 checkpoint inhibitors are the primary treatment for advanced lung cancer [93,94,95,96].

Thus, mAbs targeting either PD-1 (pidilizumab) or its ligands (durvalumab and atezolizumab) have also performed well in clinical trials [97]. In 2011, the FDA approved anti-CTLA-4 mAb Ipilimumab for melanoma patients [98].

The discovery of new immune checkpoint pathways, either blocking or activating these pathways, is an emerging key role in regulating T cell response. New investigations have reported some negative regulators of T cell activation as adjuvant cancer drugs, e.g., lymphocyte activation gene 3 (LAG3), T cell immunoglobulin 3 (TIM3), V-domain immunoglobulin suppressor of T cell activation (VISTA), B7-H3 and T cell immunoreceptor with immunoglobulin, and immunoreceptor tyrosine-based inhibitory motif domains (TIGIT) [99,100,101]. Like PD1, LAG3 is another vital checkpoint, which is closely related to CD 4 [102]. It has an inhibitory ligand that reduces T cell activation by blocking CD4 contact sites on MHC class II proteins to keep the immune system in check [103,104]. LAG-3 receptors are upregulated on both Tregs and anergic T cells [105]. TIM3 is another negative regulator of the T cell response, which is a T helper type 1-specific cell surface molecule that regulate their responses, induces peripheral tolerance, and helps in T cell exhaustion [106]. Moreover, its expression correlates with poor prognosis in non-small-cell lung cancer and follicular lymphoma, suggesting a role in cancer progression [59,107].

Clinically approved antibodies against PD-L1/PD-1 have demonstrated therapeutic efficacy across a range of human cancers [108,109,110]. In line with this note, cis-platinum (Cis-Pt)-mediated chemotherapy is significantly impaired due to induction of PD-L1 expression, enhanced drug efflux by multidrug resistance protein 1 (MDR-1) and increased tumor metastasis. Selective accumulation of metformin-modified chitosan (Ch-Met) in mitochondria can disrupt mitochondrial function, which eventually inhibits PD-L1 expression and lowered tumor metastasis. Therefore, it was demonstrated that Ch-Met could sensitize the chemotherapy efficacy of Cis-Pt [111]. Therefore, an antagonistic antibodies-mediated immunotherapeutic approach targeting PD-1 or its ligand PD-L1 are being significantly used to treat a wide range of cancer types and can substantially improve patient survival. Thus, to inhibit the expression and/or activity of PD-L1, specific small-molecule inhibitors are being produced, screened, and approved [109,112,113]. Herein, we summarize small-molecule inhibitors that have the potential to inhibit the mechanisms for regulating PD-L1 expression in Table 3.

## 8. Monoclonal Antibodies Therapies

Monoclonal antibodies (mAbs)-based immunotherapy is considered to be a main component of cancer therapy [76]. Using mAbs to target tumor cell antigens might be a more effective and less toxic treatment than traditional chemotherapy, fueling the interest in designing immunotherapies targeting tumor-specific antigens [118]. The tirst mAb target discovered was CD20, which was found to be overexpressed on cancerous B cells in non-Hodgkin’s lymphoma (NHL), but absent on healthy immature B cells. Thus, it was made specifically to work against cancerous cells using anti-CD20 mAb rituximab [119]. Therefore, targeting antigens overexpressed on solid tumors drastically increased the efficacy of mAbs. These target antigens include epidermal growth EGFR and HER2 against colorectal and breast cancers, respectively [120,121]. In addition, other targets include TGF-β, VEGF, and PDGF/PDGFR signaling. Fresolimumab is a mAb that targets TGF-β [122], bevacizumab blocks VEGF by binding to its receptor [123], ramucirumab is a mAb to VEGFR2, and icrucumab is a mAb to VEGFR1, all of which are approved for the treatment of many different cancers [124,125]. More recently, instead of targeting tumor antigens, stimulating T cell anti-tumor immunity using bispecific T Cell Engager (BiTE) antibodies to target a tumor antigen—CD19 and the activating receptor, CD3—on T cells is the first mAb approach that enhances anti-tumor capabilities [126]. Thus, the FDA approved CD19-CD3 BiTE blinatumomab to treat acute lymphoblastic leukemia patients in 2017 [127].

## 9. Immune System Modulators

Immunomodulators are a group of drugs that mainly target the pathways that treat multiple myeloma and a few other cancers [128]. Anticancer drugs, such as anthracyclines, thalidomides, lenalidomides, pomalidomide, Thalidomide (Thalomid), and hypomethylating agents (HMA) are known as immunomodulating drugs (or IMiDs), and they could also strengthen the immune system to attack cancer cells at a relatively low dose [129]. The FDA has approved lenalidomide, a derivative of Thalidomide, for maintenance therapy of posttransplant myeloma patients [130,131]. Thalidomide cause tumoricidal effects by causing cell cycle arrest and also has antiangiogenic properties [132]. Anthracyclines, e.g., doxorubicin or mitoxantrone, affect cancer cells by enhancing the cell surface expression of calreticulin (CRT) followed by the release of high-mobility group box 1 (HMGB1), ATP, annexin A1, and type I interferon from cancer cells [133,134,135,136]. HMAs impair DNA methylation by inhibiting DNA methyltransferase. Its examples are azacitidine or decitabine that have been approved for the treatment of myelodysplastic syndromes and acute myeloid leukemia [137,138].

## 10. Cytokines Therapy

Cytokines are small glycoproteins that are crucial mediating interactions between immune and nonimmune cells in the tumor microenvironment [139,140,141,142]. Some cytokines, including interleukin-2 (IL-2), IL-12, IL-15, IL-18, IL-21, GM-CSF, CCL21, and type 1 interferons, have shown to have antitumor activity in preclinical studies [142,143]. Most importantly, interleukins, and interferons are being used to treat cancer. Among which, IL-2 is one of the first cytokines that has been studied in cancer treatment. IL-2 can be used as a single drug treatment for cancer therapies, or it can be combined with other chemotherapy; for example, IL-2 fused with Diphtheria toxin proteins showed promising results in phase III trials of cutaneous T cell lymphoma (CTCL) patients [144]. A low dose of IL-2 has the advantage of preferentially expanded Tregs. Synergistic activity of IL2 and rituximab was seen in a mouse model against NHL when IL-2 was administered at a low dose. Although some early phase 1 studies showed promising results for the combination of rituximab and IL-2 [145,146], a study has demonstrated the effect of IL2 treatment on the expression of FOXP3 in CD3^+^CD25^+^ T cells and made Tregs phenotype expand during low-dose therapy through a STAT-dependent mechanism [147]. However, recent discoveries are focusing on using a combination of IL2 with Treg inhibitors, such as anti-CTLA-4 and anti-PD-1 [148].

Interferons have also been shown to be effective in cancer immunotherapy. IFN-α was used to treat low-grade indolent NHLs with lower activity [149,150] but a combination led to better results due to ADCC [151]. Type I IFNs also play an important role in the regulation of NK cell cytotoxicity [152]. IFNs are actively combined with various interferons and have also been combined with chemotherapy and mAbs for the treatment of various types of tumors [153].

## 11. Adoptive Transfer Therapy and Chimeric Antigen Receptor (CAR) T Cell Therapy-Advantages and Disadvantages

CAR T cells therapy is the most powerful immunotherapy works on boosting the patients’ own immune system to destroy the cancer. Immune cells, such as T cells, recognize foreign antigens through their receptors that allow them to attach to foreign antigens and instigate other parts of the immune system to destroy the foreign substance [93,154]. On this note, chimeric antigen receptor (CAR) T cells therapy involves T cells, and it is engineered by adding a gene for a receptor (called a chimeric antigen receptor, or CAR) that helps these changed T cells to specifically recognize TAAs. Each CAR is made for a specific cancer antigen depending on cancer types, e.g., CD19 is an antigen for leukemia or lymphoma. Therefore, the CAR T cells therapies are specifically designed to bind with the CD19 antigen to treat these cancers but not any other type of cancer [154,155,156].

CAR T cells therapy is a multi-step procedure starting from the preparation of CAR T cells to their transfer to patients (T cell collection, engineering chimeric T cells and infusing CAR T cells to the patient). Many CAR T cells therapies have been approved by the Food and Drug Administration (FDA) to treat blood cancers, lymphomas, leukemia, and—most recently—multiple myeloma [157]. There are crucial CAR T cells therapy-associated side effects that need to be addressed, such as immunosuppression with an increased risk of serious infections, nervous system problems, allergic reactions, and cytokine release syndrome (CRS) due to overexpression of cytokines into the blood, which can ramp up the immune system and the amount of life-threatening toxicities [158,159]. These underlying toxicities associated with CAR T cells therapy have been most extensively characterized in patients receiving CD19-directed CARs [160,161]. Development of tumor resistance to a single antigen targeting CAR constructs is one of the most challenging limitations of CAR T cells therapy. The downregulation/loss of CD19 antigen is 30–70% of patients who have recurrent disease after treatment [162,163]. In addition, the expression of solid tumor antigens on normal tissues at varying levels make tumor cells targeting challenging. Therefore, antigen selection is crucial in CAR design to limit “on-target off-tumor” toxicity. As can be seen in the case of first-generation CAR T cell targeting TAG72 in colorectal cancer, it showed no anti-tumor response. Thus, tumor-restricted post-translational modifications are currently being investigated to specifically target tumor cells [164,165].

## 12. Concluding Remarks

Harnessing the body’s own immune system to fight cancer can be achieved by cancer immunotherapy. By jumpstarting the immune responses in a proper manner without eliciting unwanted toxicities could help in modulating the immune system to eliminate cancer. This can be done by involving not only T cells, but also other immune cells such as antigen-presenting cells and natural killer cells. Due to getting success in clinical trials with immunotherapies and cancer vaccines, new hope has arisen to fight cancer. These strategies are increasingly tolerated than traditional chemotherapeutic agents, despite higher immune-related adverse effects. The achievement of new approaches in biological therapies make us more confident in treating cancer.

## Figures and Tables

**Table 1:** Current status of cancer clinical research on immunotherapy.

Target	Drug INN (Brand Name)	Cancer Type	Current Status	Ref.
Antibodies Based				
CTLA-4	Ipilimumab (Yervoy)	Melanoma (2011) and Renal cell carcinoma (2018)	FDA approved	[67,68,69]
		Multiple cancers	Phase I-III	[69]
	Tremelimumab (Imjudo)	Antineoplastic; liver cancer	FDA approved	[69,70]
PD-1	Nivolumab (Opdivo)	Melanoma (2014), NSCLC (2015), and Renal (2018) cancers, Hodgkin lymphoma	FDA approved	[69,71,72]
		Multiple cancers	Phase I-III	[69]
	Pembrolizumab (Keytruda)	Melanoma (2014), Various (2015)	FDA approved	[73]
		Multiple cancers	Phase I-III	[69]
	MED10680	Multiple cancers	Phase I	[69]
	AMP-224	Multiple cancers	Phase I	[69]
	Pidilizumab	Multiple cancers	Phase I-II	[69]
	Cemiplimab (Libtayo)	Cutaneous squamous-cell carcinoma (2018)	FDA approved	[69,74]
PD-L1	Atezolizumab (Tecentriq)	Bladder, NSCLC (2016), and TNBC (2019), hepatocellular carcinoma, HCC (2020)	FDA approved	[69] [74,75]
	Avelumab (Bavencio)	Urothelial Carcinoma (2017), Merkel Cell Carcinoma (2017), Renal carcinoma (2019)	FDA approved	[74]
	MED14736	Multiple cancers	Phase III	[69]
	Avelumab (Bavencio)	Merkel cell carcinoma (2017), Rena (2019), Urothelial carcinoma (2020)	FDA approved	[74]
	BMS-936559	Multiple cancers	Phase I	[69]
	Durvalumab (IMFINZI)	Bladder Cancer (2017), NSCLC (2018)	FDA approved	[69,74]
LAG-3	IMP321	Multiple cancers	Phase I	[69]
	BMS-986016	Multiple cancers	Phase I	[69]
	Relatlimab (Opdualag)	Melanoma (2022)	FDA approved	[70]
VEGF	Bevacizumab	Colorectal (2004), NSCLC (2006, 2018), Renal (2009), Cervical (2014), Glioblastoma (2009), and Ovarian (2018) Cancers	FDA approved	[74,76]
VEGF-A, Ang-2	Faricimab (Vabysmo)	wAMD, DME	FDA approved	[70]
VEGFR2	Ramucirumab (Cyramza)	Gastric cancer (2014), NSCLC (2020), HCC (2019)	FDA approved	[74,76,77]
EGFR	Cetuximab	Colorectal cancer (CRC) (2004, 2012) and Head and neck squamous cell carcinoma (2006, 2011)	FDA approved	[74,76]
	Necitumumab (Portrazza)	NSCLC (2015)	FDA approved	[74,76,77]
	Panitumumab (Vectibix)	Colorectal Cancer (2006)	FDA approved	[76]
PDGFRα	Olaratumab (Lartruvo)	Soft-tissue sarcoma (2016)	FDA approved	[76]
HER2	Pertuzumab (Perjeta)	HER2-positive Breast cancer (2012)	FDA approved	[74,76]
	Trastuzumab (Herceptin)	HER2-positive Breast cancer (1998)	FDA approved	[74,76]
	Ado-trastuzumab emtansine (Kadcyla)	HER2-Breast cancer (2013)	FDA approved	[76]
	Fam-trastuzumab deruxtecan (Enhertu)	HER2-positive Breast cancer (2019)	FDA approved	[76]
	Trastuzumab tucatinib	HER2-positive Breast cancer (2020)	FDA approved	[74]
CCR4	Mogamulizumab (Poteligeo)	Cutaneous T cell lymphoma (2018)	FDA approved	[76]
CD20	Obinutuzumab (Gazyva)	Chronic lymphocytic leukemia (2013), follicular lymphoma (2017)	FDA approved	[74,76]
	Ofatumumab (Arzerra)	Chronic lymphocytic leukemia (2014)	FDA approved	[74,76]
	Rituximab (MabThera, Rituxan)	B-Cell Lymphoma (1997)	FDA approved	[76]
	Ibritumomab tiuxetan (Zevalin)	NHL (2004)	FDA approved	[70,76]
	tositumomab Iodine-131 (Bexxar)	NHL (2003)	FDA approved	[76]
	Ublituximab	Chronic lymphocytic leukemia, CLL, non-Hodgkin’s lymphoma) and non-cancer (multiple sclerosis)	Phase III	[70,77]
CD33	Gemtuzumab ozogamicin (Mylotarg)	Acute myeloid leukemia (2000)	FDA approved	[74,76]
CD30	Brentuximab vedotin (Adcetris)	Hodgkin’s lymphoma and Anaplastic large-cell lymphoma (2011)	FDA approved	[76,78,79]
CD79B	Polatuzumab vedotin (Polivy)	Diffuse large B-cell lymphoma (2019)	FDA approved	[74,76]
CD22	Inotuzumab ozogamicin (BESPONSA)	Acute lymphoblastic leukemia (2017)	FDA approved	[74,76]
	Moxetumomab pasudotox (Lumoxiti)	Hairy-cell leukemia (2018)	FDA approved	[74,76]
CD19	Inebilizumab (Uplizna)	Neuromyelitis optica and neuromyelitis optica spectrum disorders (2022)	FDA approved	[70]
CD19, CD3	Blinatumomab (Blincyto)	Acute lymphoblastic leukemia (2014)	FDA approved	[74,76]
TROP2	Sacituzumab govitecan (Trodelvy)	TNBC (2020)	FDA approved	[70,74,76]
CD3	Muromonab-CD3 (Orthoclone Okt3)	Reversal of kidney transplant rejection (1986)	FDA approved	[77]
CD3, BCMA	Teclistamab (TECVAYLI)	Multiple myeloma (2022)	FDA approved	[70]
gp100, CD3	Tebentafusp (KIMMTRAK)	Metastatic uveal melanoma (2022)	FDA approved	[70]
CD30, CD3	Mosunetuzumab (Lunsumio)	Follicular lymphoma (2022)	FDA Review	[70]
CD38	Daratumumab (Darzalex)	Multiple Myeloma (2015)	FDA approved	[74,76]
	Isatuximab (Sarclisa)	Multiple Myeloma (2020)	FDA approved	[74,76]
GD2	Dinutuximab (Qarziba; Unituxin)	Neuroblastoma (2015)	FDA approved	[74,76]
Nectin-4	Enfortumab Vedotin (Padcev)	Bladder cancer (2019), Urothelial cancer (2022)	FDA approved	[70,74,76]

**Table 2:** Summary of small drugs inhibitors.

Small Drugs Based				
Target	Drug INN (Brand Name)	Cancer Type	Current Status	Ref.
EGFR	Gefitinib	NSCLC (2015)	FDA approved	[74,80]
	Erlotinib HCl (Tarceva)	NSCLC (2004)	FDA approved	[80]
	Osimertinib mesylate	NSCLC (2020)	FDA approved	[74,80]
	Dacomitinib (Vizimpro)	EGFR-mutated NSCLC (2018)	FDA approved	[74,80]
	Mobocertinib succinate (Exkivity)	EGFR exon 20-mutated NSCLC (2021)	FDA approved	[80]
HER2	Tucatinib (Tukysa)	HER2-positive breast cancer (2020)	FDA approved	[74,80]
EGFR, HER2, and HER4	Neratinib maleate (Nerlynx)	HER2-overexpressed breast cancer (2017)	FDA approved	[80]
	Afatinib dimaleate (Gilotrif)	Metastatic NSCLC with EGFR exon 19 deletion or exon 21 (L858R) mutation (2013)	FDA approved	[80]
PARP	Olaparib (Lynparza)	Advanced BRCA-mutated ovarian cancer (2020)	FDA approved	[74,80]
	Rucaparib camsylate (Rubraca)	BRCA-positive ovarian cancer (2016)	FDA approved	[74,80]
	Niraparib tosylate (Zejula)	Epithelial ovarian, fallopian tube, or primary peritoneal cancer (2017)	FDA approved	[80]
PDGFRα	Avapritinib (Ayvakit)	metastatic gastrointestinal stromal tumor (GIST) with platelet-derived growth factor receptor alpha (PDGFRA) exon 18 mutations (2020)	FDA approved	[74,80]
Multitarget TKI (VEGFRs, PDGFRα/β, CSF1R, KIT, and FLT3)	Sunitinib malate (Sutent)	Imatinib-resistant GIST and advanced RCC (2013)	FDA approved	[74,80]
Multitarget TKI (RET, VEGFRs, KIT, PDGFRα/β, FGFR1/2, RAF1, BRAF, and BRAF^V600E^)	Regorafenib (Stivarga)	Metastatic colorectal cancer (2012)	FDA approved	[74,80]
Multitarget TKI (VEGFR2/3, PDGFRβ, FLT3, KIT, RAF1, and BRAF)	Sorafenib toylate (Nexavar)	Advanced RCC (2005)	FDA approved	[80]
Multitarget TKI (VEGFRs, PDGFRα/β, FGFR1/2, KIT)	Pazopanib HCl (Votrient)	Metastatic RCC (2009)	FDA approved	[80]
Multitarget TKI (VEGFRs, FGFRs, PDGFRα, RET, and KIT)	Lenvatinib mesylate (Lenvima)	Thyroid cancer (2015)	FDA approved	[80]
Multitarget TKI (VEGFR2/3, EGFR, and RET)	Vandetanib (Caprelsa)	Unresectable or metastatic medullary thyroid cancer (2011)	FDA approved	[74,80]
Multitarget TKI (VEGFRs, MET, RET, FLT3, KIT, TIE2, and AXL)	Cabozantinib S-malate (Cometriq)	Progressive, metastatic medullary thyroid cancer (2012)		[80]
VEGFRs	Axitinib (Inlyta)	Advanced RCC (2012)	FDA approved	[74,80]
	Tivozanib HCl (Fotivda)	Advanced RCC (2021)	FDA approved	[80]
mTOR	Everolimus (Afinitor)	Advanced RCC (2009), HER2-negative breast cancer after failure of treatment with letrozole or anastrozole (2012), nonfunctional neuroendocrine tumors of gastrointestinal or lung origin with unresectable, locally advanced, or metastatic disease (2016)	FDA approved	[74,80]
	Temsirolimus (Torisel)	Advanced RCC (2007)	FDA approved	[80]

**Table 3:** Small drugs with checkpoint inhibition capacity in cancer immunotherapy.

Target	Drug	Cancer Type	Modulate PDL1	Ref.
Histone methyltransferase EZH2	Tazemetostat and DZNep	Prostate cancer	Transcriptional upregulation of PD-L1	[114]
	Tazemetostat (Tazverik)	Epithelioid sarcoma (2020)	Transcriptional upregulation of PD-L1 via decreased H3K27me3, FDA approved	[80]
Histone deacetylase inhibitor	Vorinostat (Zolinza)	Cutaneous T cell lymphoma (2006)	Transcriptional upregulation of PD-L1, FDA approved	[80,114]
DNA methyltransferases	Decitabine (Dacogen)	Myelodysplastic syndrome (2006)	Transcriptional upregulation of PD-L1 via decreased DNA methylation in the PD-L1 promoter region, FDA approved	[80,114]
EGFR	Gefitinib (Iressa)	NSCLC (2004), HNSCC and TNBC	Transcriptional downregulation of PD-L1, FDA approved	[80,114]
	Osimertinib mesylate (Tagrisso)	NSCLC with EGFRT790M mutations (2015)		
	Erlotinib HCL (Tarceva)	NSCLC (2003)		
JAK	Ruxolitinib phosphate (Jakafi)	Intermediate or high-risk myelofibrosis (2011)	Downregulation of PD-L1, FDA approved	[80,114]
	Fedratinib HCl (Inrebic)	Myelofibrosis (2019)	Downregulation of PD-L1, FDA approved	[80,114]
ZFP36 (Tristetraprolin)	Doxorubicin	NSCLC and breast cancers	Downregulates translation of PD-L1	[80,114,115]
AMPK	Metformin and A-769662	Multiple cancers	Increased PD-L1 phosphorylation and degradation	[80,116,117]

## Data Availability

Not applicable.
